# DNA Demethylation Rescues the Impaired Osteogenic Differentiation Ability of Human Periodontal Ligament Stem Cells in High Glucose

**DOI:** 10.1038/srep27447

**Published:** 2016-06-08

**Authors:** Zhi Liu, Tian Chen, Wenhua Sun, Zongyi Yuan, Mei Yu, Guoqing Chen, Weihua Guo, Jingang Xiao, Weidong Tian

**Affiliations:** 1 Department of Oral and Maxillofacial Surgery, Hospital of Stomatology, Southwest Medical University, Luzhou 646000, P.R. China; 2 National Engineering Laboratory for Oral Regenerative Medicine, West China Hospital of Stomatology, Sichuan University, Chengdu 610041, P.R. China; 3 State Key Laboratory of Oral Diseases, West China Hospital of Stomatology, Sichuan University, Chengdu 610041, P.R. China; 4 Department of Orthodontics, West China School of Stomatology, Sichuan University, Chengdu 610041, P.R. China; 5 Orofacial Reconstruction and Regeneration Laboratory, Hospital of Stomatology, Southwest Medical University, Luzhou 646000, P.R. China; 6 Department of Oral and Maxillofacial Surgery, West China School of Stomatology, Sichuan University, Chengdu 610041, P.R. China

## Abstract

Diabetes mellitus, characterized by abnormally high blood glucose levels, gives rise to impaired bone remodeling. In response to high glucose (HG), the attenuated osteogenic differentiation capacity of human periodontal ligament stem cells (hPDLSCs) is associated with the loss of alveolar bone. Recently, DNA methylation was reported to affect osteogenic differentiation of stem cells in pathological states. However, the intrinsic mechanism linking DNA methylation to osteogenic differentiation ability in the presence of HG is still unclear. In this study, we found that diabetic rats with increased DNA methylation levels in periodontal ligaments exhibited reduced bone mass and density. *In vitro* application of 5-aza-2′-deoxycytidine (5-aza-dC), a DNA methyltransferase inhibitor, to decrease DNA methylation levels in hPDLSCs, rescued the osteogenic differentiation capacity of hPDLSCs under HG conditions. Moreover, we demonstrated that the canonical Wnt signaling pathway was activated during this process and, under HG circumstances, the 5-aza-dC-rescued osteogenic differentiation capacity was blocked by Dickkopf-1, an effective antagonist of the canonical Wnt signaling pathway. Taken together, these results demonstrate for the first time that suppression of DNA methylation is able to facilitate the osteogenic differentiation capacity of hPDLSCs exposed to HG, through activation of the canonical Wnt signaling pathway.

Alveolar bone surrounding the roots of teeth is characterized by high plasticity and is considered to be the most active tissue in human bone metabolism. The remodeling of alveolar bone is not only impacted by tooth development and masticatory pressure, but also stimulated by tooth movement in response to orthodontic treatment^1^. Interestingly, alveolar bone turnover is affected by periodontal ligament stem cells (PDLSCs), which are able to differentiate into osteoblasts, adipocytes, neurons, cementoblast-like cells, and collagen-forming cells *in vivo*^2^. Under physiological conditions, osteoblasts and osteoclasts are responsible for maintaining homeostatic control of alveolar bone generation^3^.

However, this balance can be disrupted in pathologic circumstances. Diabetes mellitus (DM), which gives rise to high blood glucose levels, is a heterogeneous group of disorders^4^. Recent evidence suggests that alteration of bone metabolism is a common complication of DM. DM is considered as a risk factor for periodontitis, in which alveolar bone loss is one of the main outcomes^5^. In the presence of high concentrations of glucose, the number of bone-forming cells rapidly drops^6^ and the expression of transcription factors that regulate osteogenesis is blocked^7^. Meanwhile, elevated inflammatory mediators contribute to an increase in cell apoptosis, which gives rise to osteoporosis and reduced osseous healing^8^,^9^. Despite the similar clinical manifestations and the close pathological connection between diabetes and periodontitis, the underlying molecular mechanism is obscure.

Epigenetic regulation of gene expression is known to be associated with diverse cellular processes such as cell stemness, development, and differentiation^10^. DNA methylation, a form of epigenetic modification, is mediated by DNA methyltransferases (DNMTs) which frequently modify cytosine at position 5 in CpG dinucleotides to create 5-methylcytosine (5-mC)^11^,^12^,^13^. In particular, DNA methyltransferase 1 (DNMT1), DNA methyltransferase 3a (DNMT3a) and DNA methyltransferase 3b (DNMT3b) are considered to play key roles in the process of mammalian DNA methylation^14^,^15^,^16^. It has been widely reported that methylation in promoter regions of genes negatively influence the gene’s transcription^17^,^18^. Therefore, DNA hypermethylation of CpG islands is correlated with gene silencing. Moreover, methylation is an epigenetic regulator that does not alter the DNA sequence and is known to be reversible^19^. Several studies have shown that 5-aza-2′-deoxycytidine (5-aza-dC), a widely used DNA demethylating agent, prevents methylation by inhibiting the maintenance methyltransferase DNMT^20^, thereby facilitating the differentiation of mesenchymal stem cells (MSCs) into osteoblasts by up-regulating the expression of osteogenic genes^21^,^22^. Nonetheless, whether 5-aza-dC could enhance the osteogenic differentiation potential of human (h) PDLSCs by reducing the levels of DNA methylation under high glucose (HG) conditions remains to be elucidated.

Here, we evaluated the levels of DNA methylation and the degree of alveolar bone loss in a diabetic rat model. Additionally, hPDLSCs were isolated and treated with 5-aza-dC in the presence of HG. The effects of 5-aza-dC were subsequently evaluated in terms of DNA methylation levels, alkaline phosphatase (ALP) activity, formation of calcified nodules and the expression of osteogenic-related markers (*ALP*, osteocalcin [*OCN*], osteopontin [*OPN*] and osterix [*OSX*]). Furthermore, we found that the canonical Wnt signaling pathway was involved in the 5-aza-dC-enhanced osteogenic potential of hPDLSCs. In this study, we show for the first time that 5-aza-dC rescues the attenuated potential of HG treated hPDLSCs to differentiate into osteocytes, and that this rescue was mediated by the canonical Wnt signaling pathway. This suggests that 5-aza-dC can be used as a potential treatment for periodontal ligament regeneration in diabetic patients.

## Results

### Diabetic rats with increased DNA methylation levels in periodontal ligaments exhibit reduced bone mass and density

**Figure 1:**
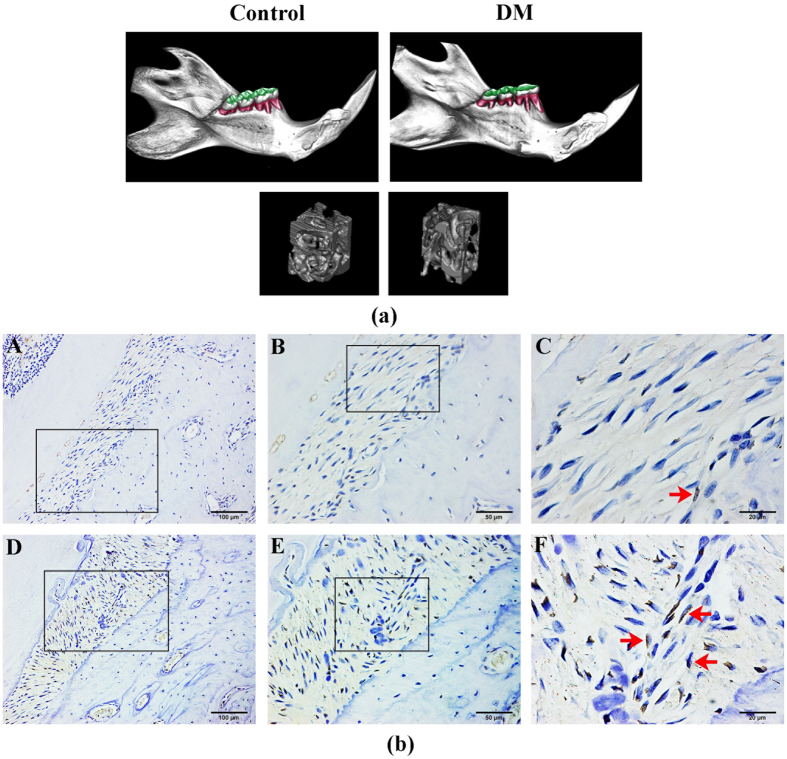
Diabetic rats with increased DNA methylation levels in the periodontal ligament exhibit reduced alveolar bone mass and density. (**a**) Reduced alveolar bone mass and density in microCT images of 18-week-old diabetic rats (right) compared with control rats. (**b**) Immunohistochemical analysis of 5-mC expression in 18-week-old diabetic rat periodontal ligament tissues (A–C) compared with control group (D–F). Red arrows show positive staining of 5-mC expression in rat periodontal ligament sections.

To study the effect of HG levels on osteogenesis in hPDLSCs, we first collected mandibular bones from 18-week-old diabetic rats. As shown in Fig. 1a, microCT analysis (details in S2 Table) demonstrated a marked reduction in bone mass as well as trabecular bone volume in alveolar bones of diabetic rats compared with control littermates. This suggested impairment in the osteogenic capacity of the periodontal ligaments in diabetic rats. Next, to assess the DNA methylation status of rat periodontal ligaments, we measured 5-mC by immunohistochemistry at postnatal week 18. Notably, analysis of histological sections revealed that 5-mC expression was obviously higher in diabetic rats compared with normal littermates (Fig. 1b). These data were consistent with clinical observations and suggested that cells exposed to HG may result in a reduction in bone mass and density by increasing the levels of DNA methylation in periodontal ligament tissues.

### Characterization of hPDLSCs and examination of DNMT1 expression in hPDLSCs

**Figure 2:**
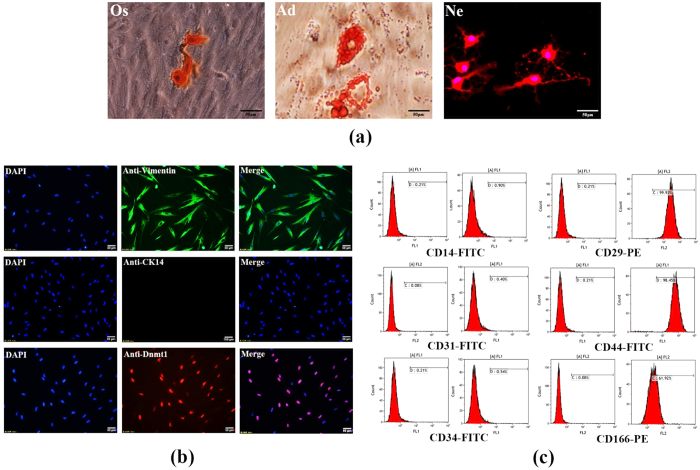
Characterization of hPDLSCs and examination of DNMT1 expression in hPDLSCs. Immunocytochemical analysis showed that isolated hPDLSCs were negative for CK14 and positive for vimentin. Additionally, DNMT1 was localized to the nuclei and abundantly expressed in hPDLSCs (**a**). Flow cytometric analysis showed that cultured hPDLSCs were negative for CD14 (0.90%), CD31 (0.40%) and CD34 (0.54%) but positive for CD29 (99.92%), CD44 (98.45%) and CD166 (61.92%). Mouse IgG isotype control antibodies conjugated to FITC or PE were used as negative controls (**b**). After being separately cultured in osteogenic (Os) or adipogenic (Ad) medium for 21 days, mineralized nodules were stained with alizarin red solution and oil droplets were stained with oil red O solution. hPDLSCs that were cultured in neurogenic (Ne) media for 2 hours formed axon-like structures (**c**).

In order to elucidate the underlying mechanism of these *in vivo* observations, we utilized hPDLSCs, which were harvested from the premolars of orthodontic patients, to further investigate the relationship between HG and DNA methylation in human periodontal ligaments. Immunofluorescence revealed that these isolated cells were negative for the epithelial cell marker CK14, but positive for the mesenchymal cell marker vimentin. Moreover, *DNMT1* was detected in hPDLSCs showing the nuclear localization (Fig. 2a). Similarly, flow cytometric analysis demonstrated that mesenchymal stem cell markers^23^ (CD29, CD44 and CD166) were highly expressed, while the mononuclear marker (CD14), the megakaryocyte marker (CD31) and the hematopoietic marker (CD34) were expressed at low levels (Fig. 2b). Furthermore, culture experiments suggested that the cells were capable of differentiating into osteoblasts, adipocytes, and nerve-like cells *in vitro* (Fig. 2c). Taken together, these results showed that the isolated hPDLSCs exhibited MSC characteristics and multi-lineage differentiation ability.

### HG increases the DNA methylation level in hPDLSCs

**Figure 3:**
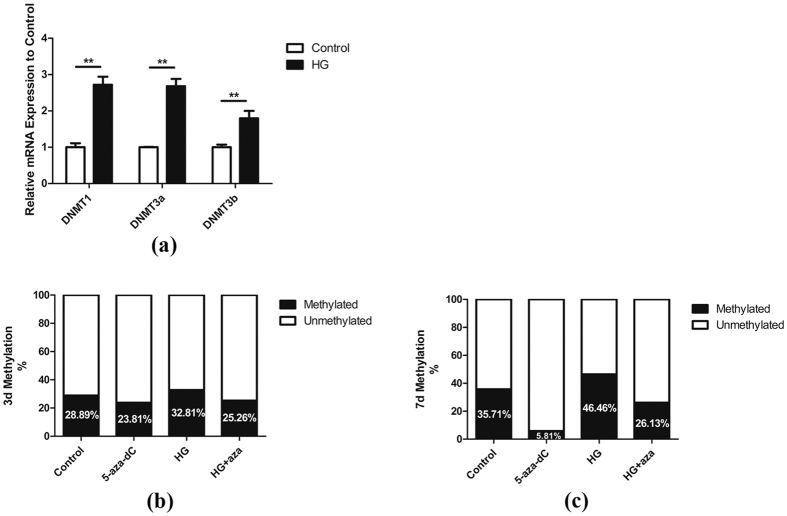
HG increases the DNA methylation levels of hPDLSCs. (**a**) Human periodontal ligament stem cells (hPDLSCs) were treated with glucose at a concentration of 30 mM (HG) for 4 days, and then mRNA levels of DNA methyltransferases *DNMT1*, *DNMT3a* and *DNMT3b* were assessed relative to the control (without HG exposure) using quantitative RT-PCR analysis (n = 3). *GAPDH* served as internal control. *p < 0.05, **p < 0.01 (Student’s t-test). hPDLSCs were cultured in different groups as indicated for 3 (**b**) or 7 (**c**) days. Total genomic DNA was extracted from the hPDLSCs and global DNA methylation levels were measured.

To address the possibility that high concentrations of glucose may result in increased DNA methylation levels, cells were exposed to either 5.5 mM (Control, normal glucose) or 30 mM glucose (high glucose: HG) for 4 days, and the relative mRNA expression of *DNMT1*, *DNMT3a* and *DNMT3b* was analyzed using quantitative RT-PCR. As shown in Fig. 3a, treatment with HG resulted in a significant increase in expression of DNMTs. Moreover, global DNA methylation analysis at days 3 and 7 showed that cells incubated with HG exhibited hypermethylation compared with the control group (Fig. 3b,c). At the same time, we evaluated the effect of 5-aza-dC on the reduction of DNA methylation levels. We found that both in the control group and the HG group, treatment of cells with 1 μM 5-aza-dC led to a reduction in methylation (Fig. 3b,c).

### 5-aza-dC rescues the osteogenic differentiation capacity of hPDLSCs under HG conditions

**Figure 4:**
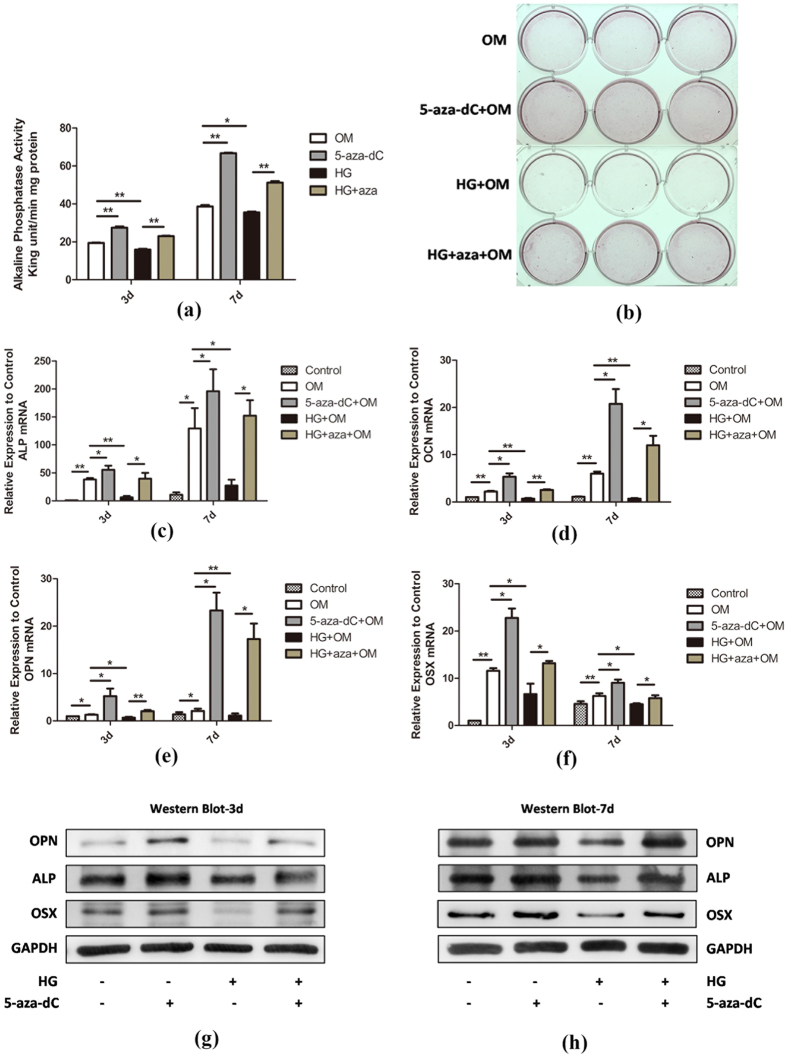
5-aza-dC rescues the osteogenic differentiation capacity of hPDLSCs under HG conditions. (**a**) hPDLSCs were incubated in osteogenic medium (OM) with 30 mM glucose (HG) or 1 μM 5-aza-dC for 3 or 7 days, and then ALP activity was measured (n = 3). The values (King unit/min/mg protein) are expressed as the mean ± SD. *p < 0.05, **p < 0.01 (Student’s t-test). (**b**) hPDLSCs were incubated in osteogenic medium (OM) for 2 weeks and mineralization of different groups as indicated was confirmed using alizarin red staining. (**c**–**f**) hPDLSCs were incubated in osteogenic medium (OM) with 30 mM glucose (HG) or 1 μM 5-aza-dC for 3 or 7 days, and then mRNA levels of osteogenic markers *ALP* (**c**), *OCN* (**d**), *OPN* (**e**) and *OSX* (**f**) were subjected to quantitative RT-PCR analysis (n = 3). *GAPDH* served as internal control. *p < 0.05, **p < 0.01 (Student’s t-test). (**g**,**h**) hPDLSCs were incubated in osteogenic medium (OM) with 30 mM glucose (HG) or 1 μM 5-aza-dC for 3 (**g**) or 7 (**h**) days, and then protein levels of ALP, OPN and OSX were measured by Western blot analysis.

Because the levels of DNA methylation were reduced by 5-aza-dC in hPDLSCs, we then explored the effect of 5-aza-dC on the osteogenic competence of hPDLSCs. To address the possibility that the matrix mineralization of hPDLSCs was enhanced by 5-aza-dC, alkaline phosphatase (ALP) activity and alizarin red staining were carried out to assess the degree of osteogenesis in hPDLSCs after differentiation for 3 or 7 days in the presence of osteogenic differentiation medium. Cells cultured in the presence of 1 μM 5-aza-dC exhibited robust ALP activity compared with the control group at day 3, indicating an increase in matrix mineralization of hPDLSCs, which was further elevated at day 7 (Fig. 3a). In contrast, hPDLSCs incubated with 30 mM glucose exhibited significant suppression of osteogenesis at days 3 and 7 (Fig. 4a). Similarly, alizarin red staining showed that treatment of cells with 5-aza-dC resulted in substantially higher levels of staining than in the control group, whereas HG conditions resulted in reduced mineralization nodule formation by hPDLSCs (Fig. 4b). Interestingly, exposure to 5-aza-dC rescued the osteogenic differentiation capacity of hPDLSCs under HG conditions (Fig. 4a,b), demonstrating that 5-aza-dC counteracted the HG-induced inhibitory effect of the hPDLSC mineralization process.

Subsequently, we assessed the mRNA and protein expression levels of osteogenic-associated genes to investigate the role of 5-aza-dC in osteogenesis in hPDLSCs. The mRNA expression levels of osteogenic markers *ALP*, *OCN*, *OPN* and *OSX* were determined using quantitative RT-PCR analysis at day 3 and 7. Consistent with the above results, treatment with 5-aza-dC led to higher expression levels of these osteogenic-related genes than that in control. Furthermore, cells exposed to HG showed a significant decrease in expression compared with other groups, and 5-aza-dC administration resulted in recovered expression levels under HG conditions (Fig. 4c–f). In addition, Western blot analysis confirmed the expression changes of OPN, ALP and OSX at the protein level (Fig. 4g,h).

### The canonical Wnt signaling pathway is involved in 5-aza-dC-induced osteogenic differentiation of hPDLSCs under normal and HG conditions

**Figure 5:**
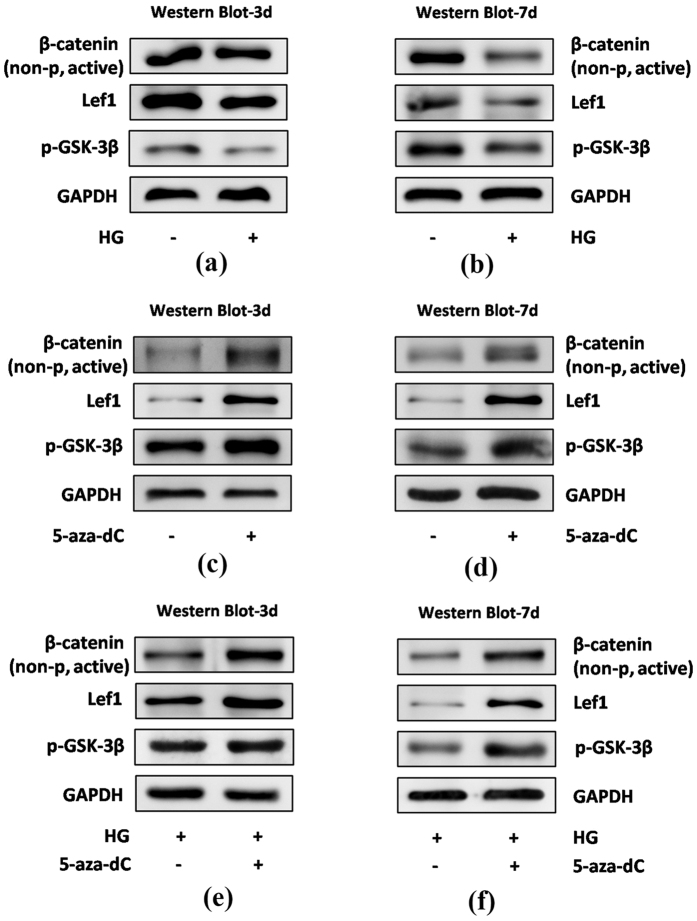
The canonical Wnt signaling pathway is involved in 5-aza-dC-induced osteogenic differentiation of hPDLSCs under normal or HG conditions. hPDLSCs were incubated in osteogenic medium under the conditions indicated, followed by the measurement of protein levels of β-catenin (non-p active), p-GSK-3β and Lef1 by Western blot analysis: (**a**,**b**) hPDLSCs were incubated with 30 mM glucose (HG) for 3 (**a**) or 7 (**b**) days; (**c,d**) hPDLSCs were incubated with 1 μM 5-aza-dC for 3 (**c**) or 7 (**d**) days; (**e,f**) hPDLSCs were incubated with 1 μM 5-aza-dC in the presence of 30 mM glucose (HG) for 3 (**e**) or 7 (**f**) days.

The canonical Wnt signaling pathway plays a crucial role in osteogenesis. Therefore, we next chose to investigate the effect on Wnt/β-catenin signaling of 5-aza-dC-induced osteogenic differentiation of hPDLSCs. When hPDLSCs were incubated with osteogenic medium for 3 or 7 days under normal or HG conditions, the protein levels of non-phospho (active) β-catenin (Ser33/37/Thr41), p-GSK-3β (Ser9) and Lef1 were detected by Western blot analysis. As shown in Fig. 5a,b, hPDLSCs exposed to 30 mM glucose showed reduced activity of the canonical Wnt signaling pathway compared with control groups at day 3 and 7. Furthermore, the expression of these Wnt-related proteins was increased significantly by 5-aza-dC administration compared with control cells (Fig. 5c,d). This was also observed in the presence of HG (Fig. 5e,f), suggesting that canonical Wnt signaling is activated in 5-aza-dC-induced osteogenic differentiation of hPDLSCs.

**Figure 6:**
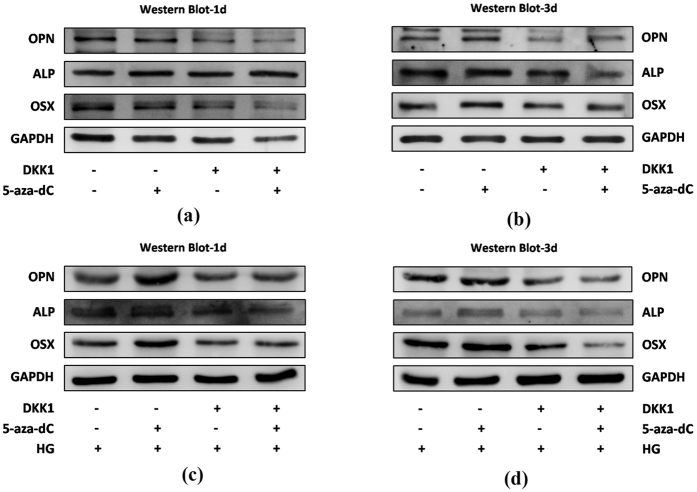
Canonical Wnt signaling pathway inhibitor DKK1 attenuates the osteogenesis of hPDLSCs with 5-aza-dC. (**a**,**b**) hPDLSCs were incubated in osteogenic medium (OM) in different groups for 1 (**a**) or 3 days (**b**), and then levels of osteogenic-related proteins, ALP, OPN and OSX, were measured by Western blot analysis. (**c,d**) hPDLSCs were incubated in osteogenic medium (OM) in the presence of 30 mM glucose (HG) in different groups for 1 (**c**) or 3 days (**d**), and then osteogenic-related protein levels of OPN, ALP and OSX were measured by Western blot analysis.

To further verify the involvement of canonical Wnt signaling in regulation of osteogenesis in hPDLSCs by 5-aza-dC, we used rhDKK1 to inhibit canonical Wnt signaling pathway activity. Western blot analysis showed that, in the presence of rhDKK1, 5-aza-dC-induced expression levels of osteogenic differentiation-related proteins were decreased (Fig. 6a,b). This was also observed in the presence of HG (Fig. 6c,d). Together these results indicate that 5-aza-dC promotes osteogenic differentiation of hPDLSCs via the canonical Wnt signaling pathway.

## Discussion

Diabetes mellitus is a complex disease, characterized by dysregulation of glucose metabolism, resulting in a range of complications, including a mass of oral complications^24^. The periodontium and alveolar bone are targets for diabetic damage in which the metabolism and cell function are severely impaired^25^. Thus, an understanding of the underlying mechanism that links DM-induced HG conditions and impaired osteogenic differentiation capacity of hPDLSCs is urgently needed. In this study, we demonstrated that the osteogenic differentiation capacity of hPDLSCs was reduced under HG conditions, and, simultaneously, DNA methylation levels were enhanced. Treatment with 5-aza-dC effectively alleviated the inhibitory effect of HG on osteogenic differentiation in hPDLSCs. Furthermore, 5-aza-dC stimulated the canonical Wnt signaling pathway to improve the osteogenic differentiation capacity of hPDLSCs.

The relationship between periodontal disease and DM has been extensively studied. On one hand, some researchers have reported that the severity of periodontal disease is greater among diabetics than in non-diabetics^26^, suggesting that this a result of a diabetes-induced microenvironment that alters immune cell function^27^, collagen and lipid metabolism^28^, and wound healing^29^. On the other hand, there is evidence to support periodontal diseases having an adverse effect on glycemic control^25^. Periodontal infection can induce a proinflammatory state, including the production of cytokines such as TNF-α and IL-6, which provides a link between periodontal disease and diabetes^30^,^31^. Our *in vivo* study using diabetic rats revealed a remarkable decrease in alveolar bone mass and density that mimics the typical clinical manifestation of DM in the periodontium. In addition to up-regulated DNA methylation levels evaluated by 5-mC expression, the width of the periodontal ligament and bone lacuna were both increased. These results confirm that diabetes causes adverse effects on alveolar bone remodeling and periodontium metabolism. Diabetes is due to either the pancreas not producing enough insulin or the cells of the body not responding properly to the insulin produced. Based on this, there are two main types of DM including Type 1 diabetes which results from the pancreas’s failure to produce enough insulin and Type 2 diabetes which is characterized by insulin resistance, a condition in which cells fail to respond to insulin properly^27^. The DM patients show the classic symptoms of weight loss, polyuria, polyphagia and polydipsia which happen in both types of DM with discrepant speed of pathogenicity. Symptoms may develop rapidly in Type 1 diabetes, while they usually develop much more slowly and may be subtle or absent in Type 2 diabetes^30^. In our research, we only introduced Type 1 diabetes in rats and therefore do not accurately recreate all aspects of the more common Type 2 diabetes in our work, still, the HG-induced cell micro- environment can simulate the hyperglycemia that happens in both types of DM and provide a solid basis to better understand the molecular mechanism of DM pathogenesis.

It has been widely reported that DNA methylation levels are increased under HG conditions. In a recent study, Ishikawa *et al.* reported that long-term exposure of pancreatic beta cells to a HG state increased DNA methylation of the insulin gene (*Ins1*) promoter at the CRE site that is important for insulin gene transcription^32^. Seman *et al.* also showed that increased DNA methylation of the promoter of the SLC30A8 gene, the product of which is essential for the transportation of zinc into the insulin-secretory granules and the subsequent crystallization of hexameric insulin, is associated with type 2 diabetes^33^. Hypermethylation in response to HG occurs not only in insulin secretion-related tissues, but also other types of cells. For example, Toperoff *et al.* found that the up-regulated DNA methylation of a prespecified regulatory site in peripheral blood leukocytes (PBLs) is associated with impaired glucose metabolism^34^. In our study, hPDLSCs cultured in HG medium expressed high levels of DNMTs (*DNMT1*, *DNMT3a* and *DNMT3b*) and exhibited increased global DNA methylation *in vitro*. Furthermore, treatment with 5-aza-dC dramatically attenuated DNA methylation levels under HG conditions, and this down-regulation was much more obvious at day 7 compared with day 3. The plausible explanation for this phenomenon is that HG-induced hypermethylation occurs in both a time-dependent and concentration-dependent manner^32^.

We reported that hPDLSCs incubated with HG resulted in a significant inhibition of osteogenesis and 5-aza-dC rescued the osteogenic differentiation capacity under HG conditions. Indeed, increased DNA methylation levels blocked the osteogenic differentiation ability and down-regulated the expression of several osteogenesis-related genes (*ALP*, *OCN*, *OPN* and *OSX*) of hPDLSCs. DNA methylation has been a growing focus of epigenetic studies of MSC osteogenic differentiation^35^,^36^,^37^. As a characteristic marker of the osteoblast phenotype, ALP is important during the early stage of osteogenic differentiation. A recent study has reported that 5-aza-dC increased the expression and activity of ALP and reduced the DNA methylation levels of the *ALP* promoter region in vascular smooth muscle cells^38^. Moreover, Delgado-Calle *et al.* showed that epigenetic methylation of CpG dinucleotides plays an important role in the regulation of ALPL expression in cells of the osteoblastic lineage and specifically in gene silencing during the transition from osteoblasts to osteocytes^39^. OSX is a zinc-finger-containing transcription factor that regulates the differentiation of pre-osteoblasts into fully functional osteoblasts^40^. 5-aza-dC-induced DNA hypomethylation resulted in expression of the *OSX* gene in myoblast C2C12 cells accompanied by the expression of the osteoblast markers ALP and OCN^41^. OPN expression in bone is predominantly seen in osteoblasts and osteocytes, but also in osteoclasts, which means that it holds a dual function in bone metabolism^42^. At the same time, OSX is required for OPN expression by binding to its promoter and up-regulating its transcription^40^. OCN, secreted solely by osteoblasts, is thought to play a crucial role in osteogenic differentiation and mineralization at the late stages of bone formation^43^. DNA methylation may also contribute indirectly to OCN transcriptional control in osteoblasts by maintaining a highly condensed and repressed chromatin structure^23^. The expression of almost all of the osteogenic-related genes we investigated in our study is altered by DNA methylation, supporting the plausibility of the observation that 5-aza-dC can rescue the osteogenic differentiation capacity of hPDLSCs under HG-induced hypermethylation conditions.

In our study, we found that the expression of non-phospho (active) β-catenin (Ser33/37/Thr41), phosphorylated GSK-3β (Ser9) and lymphoid enhancer-binding factor-1 (Lef1) was reduced in the presence of HG and increased in 5-aza-dC-treated hPDLSCs compared with control groups. Furthermore, treatment with 5-aza-dC reversed the expression of those proteins in the presence of HG. To our knowledge, this is the first time that HG-induced hypermethylation has been linked to the canonical Wnt signaling pathway in hPDLSCs. The activation of the canonical Wnt signaling pathway in DNA methylation-regulated metabolism has been widely discussed. Cho *et al.* has shown that 5-aza-dC demethylates the promoters of *Bmp2* and *Alp* thus rendering them responsive to Wnt3a, which facilitates the trans-differentiation of nonosteogenic mesenchymal cells into osteoblasts^44^. Interestingly, not only the Wnt-related ligands, but also negative regulators of the Wnt signaling pathway, including sclerostin^45^,^46^, secreted frizzled-related proteins (SFRP1/2)^47^ as well as Dickkopf-1 (DKK1)^48^ are associated with DNA methylation associated diseases and abnormalities. In this study, we showed that under HG conditions, 5-aza-dC could up-regulate the osteogenic differentiation capacity of hDPLSCs via the canonical Wnt signaling pathway, as rhDKK1, a potent antagonist of the canonical Wnt signaling pathway, inhibited this response. Future experiments using stabilized virus infection of hPDLSCs to selectively inhibit or activate the Wnt signaling pathway will shed further light on this point. In stomatology, not only the hard tissues injury like alveolar bone absorption and dentin destruction was caused by periodontitis and dental caries, but also the soft tissues injury was generated by candidiasis connecting with diabetes mellitus. Thus, our findings not only bring new perspective for thoroughly understanding the osteogenic differentiation ability of hPDLSCs in HG together with the regulation of DNA methylation, but also contribute to laying a solid foundation for further researches which focus on other oral disease related to DM and may render 5-aza-dC treated odontogenic cells as potential seed cells for DM-related disease therapy.

In summary, our study affords new insight into the mechanisms by which DNA methylation acts as a core player in the differentiation of hPDLSCs under HG conditions. Our data indicate a role for the canonical Wnt signaling pathway, activated during osteogenic differentiation and manipulated by 5-aza-dC. Our work not only contributes to our understanding of the impaired osteogenic differentiation capacity of hPDLSCs in a pathological state, but also points to novel strategies to regulate osteogenesis and periodontal ligament regeneration in clinical practice for patients with diabetes.

## Methods

All experiments were conducted in accordance with the ethical protocol approved by the Committee of Ethics of the Sichuan University. In addition, for investigations involving human subjects, informed consent has been obtained from the participants involved. All the methods were carried out in accordance with the approved guidelines.

### Animal model of diabetes

The Ethics Committee of West China College of Stomatology (Sichuan University, Chengdu, China) approved the animal experimental protocol and procedures performed. To induce diabetes, 8-week-old male Sprague-Dawley rats (weight: 220–250 g) were intraperitoneally (i.p.) injected with 1% (w/v) streptozotocin (STZ) 60 mg/kg (Sigma-Aldrich, St. Louis, MO, USA) dissolved in citrate buffer (pH 4.0–4.4) as described previously^49^,^50^. Control animals were treated with a vehicle citrate buffer (1 mL/kg). To ensure DM was established, the fasting plasma glucose (FPG) of rats was measured 2 days after STZ injection and confirmed every 3 days with an autoanalyser (Roche, Mannheim, Germany). Only animals with blood glucose levels higher than 16.7 mmol/L were considered indicative of successful diabetes induction and were included into the study.

### MicroCT analysis

Mandibles were dissected from diabetic rats and non-diabetic littermates at week 18. Samples were fixed with 4% paraformaldehyde and analyzed using Scanco Medical CT-40 (SCANCO Medical, Switzerland) instruments. The sections of alveolar bone were located at root furcartion, a place where deprived the image interference of cortical bone.

### Immunohistochemistry

Mandibles were dissected from diabetic rats and non-diabetic littermates at week 18. Immunohistochemical analyses of the samples were performed using the streptavidin-biotin complex method according to the manufacturer’s recommended protocol. The samples were then incubated with mouse polyclonal anti-5-methylcytosine (5-mC) (1:400, Abcam, UK). The sections were then stained using a 3,3′-diaminobenzidine DAB kit (ZLI-9018, ZSGB-BIO, China). Stained sections were visualized under a light microscope (Olympus BX43F; JEOL, Tokyo, Japan).

### Cell culture and treatment with reagents

Healthy premolars were extracted from patients (n = 12, 14–18 years of age, details please see Supplementary Information) with written consent signed by parents during orthodontic treatment in the West China Stomatology Hospital. All experiments were conducted in accordance with the ethics protocol approved by the Committee of Ethics of the Sichuan University. Periodontal ligaments, collected from the middle third of the root, were cultured in alpha-Modified Eagle’s Medium (α-MEM; Hyclone, USA) supplemented with 10% fetal bovine serum (FBS) (Hyclone, USA), 1% penicillin/streptomycin (Solarbio, China) in a humidified atmosphere at 37 °C with 5% CO_2_ as previously described^2^. Cell culture medium was refreshed every two days and all experiments were carried out with passage 3–4 cells. For the HG treatment, D-Glucose (Sigma-Aldrich, USA) was added to the culture medium (30 mM final concentration) for the described time intervals^51^,^52^. 5-aza-dC (Selleck Chemicals, UH, USA) was added to the culture (1 μM final concentration) for the described time intervals. rhDKK1 (R&D, Wiesbaden, Germany) was added to the culture (100 ng/ml final concentration)^53^ for 2d to block the Wnt signaling pathway.

### Multipotential differentiation capacity of hPDLSCs

Isolated hPDLSCs at passage 4 were identified by immunofluorescence. hPDLSCs (5 × 10^5^ cells) were fixed with 4% polyoxymethylene for 15 min and subsequent steps were performed according to the manufacturer’s recommendations. Antibodies used in immunofluorescent staining included antibody against vimentin (1:400, Thermo, USA) and cytokeratin (1:400; Abcam, UK). All samples were examined under a fluorescence microscope (Leica DMI 6000, Germany). Flow cytometric analysis of specific surface antigens was also used to characterize the cultured cells. hPDLSCs (5 × 10^6^ cells) were harvested and incubated with various combinations of the following fluorochrome-conjugated mouse anti-human antibodies: CD14-APC, CD29-PE, CD31-FITC, CD34-FITC, CD44-FITC and CD166-PE (BD Biosciences, USA) for 20 min at room temperature in the dark. The corresponding mouse IgG isotype control antibodies conjugated to FITC and PE were employed as negative controls in each experiment. Flow cytometry was carried out using the Beckman Coulter Cytomics FC 500 MPL system (Beckman Coulter, USA).

hPDLSCs (2 × 10^4^ cells) were loaded in 6-well plates and exposed to osteogenic medium (α-MEM supplemented with 10% FBS, 5 mM β-glycerophosphate, 100 nM dexamethasone, and 50 mg/ml ascorbic acid) or adipogenic medium (α-MEM supplemented with 10% FBS, 2 mM glutamine, 100 μM ascorbic acid, 0.5 mM isobutylmethylxanthine, 0.5 mM hydrocortisone and 60 mM indomethacin) when the cells had reached 80% confluency. After incubation for 21 days, cells were washed twice with PBS and fixed in 4% paraformaldehyde for 30 min, then incubated with either alizarin red (Sigma-Aldrich, USA) or 0.3% Oil Red O (Sigma-Aldrich, USA) for 15 min. The cells were routinely observed and visualized under a light microscope and imaged using phase-contrast inverted microscope (Nikon, Japan). For neurogenic differentiation, hPDLSCs (5 × 10^4^ cells) were routinely seeded in 6-well plates and treated with neurogenic medium (α-MEM supplemented with 10% FBS, 2 mM glutamine, 100 U/ml penicillin/streptomycin, 2% dimethylsulfoxide, 200 μM butylated hydroxyanisole, 25 mM KCl, 2 mM valproic acid sodium salt, 10 mM forskolin, 1 mM hydrocortisone and 5 μg/ml insulin). After incubation for 2 hours, cells were washed twice with PBS and fixed in 4% paraformaldehyde for 15 min. The samples were analyzed by immunofluorescence with anti-βIII-tubulin (1:200; Millipore, USA). Subsequent steps were performed according to the manufacturer’s recommendations and examined under a fluorescence microscope (Leica DMI 6000, Germany).

### Quantitative RT-PCR analysis

After treated with 5-aza-dC (1 μM), HG (30 mM) and rhDKK1 (100 ng/ml) for different periods of time, hPDLSCs (2 × 10^6^ cells) were isolated by differential digestion using trypsin/EDTA (Millipore, USA) prior to total RNA extraction using RNAiso plus (Takara, Dalian, China). cDNA synthesis was performed with SYBR® Premix Ex Taq II (Perfect Real Time kit; Takara, Dalian, China). Experiments were performed in triplicate according to the manufacturer’s instructions. Sequences of the gene-specific primers synthesized by Sangon Biotech (Shanghai, China) are listed in Table 1. Quantitative RT-PCR reaction conditions were: 95 °C for 30 s; followed by 40 cycles of 95 °C for 5 s, 60 °C for 30 s; then one cycle of 95 °C for 15 s, 60 °C for 60 s, 95 °C for 15 s. The results were analyzed using the 2^−∆∆CT^ relative quantitative method, with *GAPDH* as an internal control. Experiments were repeated three times.

### Cell DNA extraction and global DNA methylation level quantification

After being treated with 5-aza-dC (1 μM), HG (30 mM) or 5-aza-dC + HG, hPDLSCs (2 × 10^6^ cells) were detached by trypsinization and collected for DNA extraction using FitAmp™ Blood and Cultured Cell DNA Extraction Kit (Epigentek, NY, USA) according to the manufacturer’s instructions. Quantification of the global DNA methylation levels in each of the different groups, and appropriate positive and negative controls, was performed with the Methylamp™ Global DNA Methylation Quantification Ultra Kit (Epigentek, NY, USA) according to the manufacturer’s instructions. The absorbance of final processed samples was evaluated on a microplate reader at 450 nm.

For simple calculation of DNA methylation, use the following formula (the amount of the positive control is 10 ng and sample DNA is 100 ng):


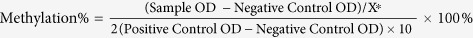
 (*X is the GC content of the species DNA)

### Alkaline phosphatase (ALP) activity

hPDLSCs were grown in α-MEM containing 5-aza-dC (1 μM), HG (30 mM) or 5-aza-dC + HG. At day 3 and 7, ALP activity of treated and untreated hPDLSCs (2 × 10^6^ cells) was determined according to the manufacturer’s recommendations with an ALP kit (Jiancheng-Nanjing, China) and normalized using protein concentration. The absorbance of each well was determined using a microplate reader at 520 nm. Enzyme activity was expressed as King unit/min/mg protein.

### Western blot analysis

hPDLSCs (2 × 10^6^ cells) were isolated by differential digestion and Western blotting was conducted according to the manufacturer’s instructions. The primary antibodies were anti-OPN (1:1000; Abcam, UK), anti-ALP (1:1,000; Abcam, UK), anti-OSX (1:1,000; Abcam, UK), anti-non-phospho (active) β-catenin (1:1000; CST, USA), anti-p-GSK-3β (1:1000; CST, USA), anti-Lef1 (1:1000; Abcam, UK) and anti-GAPDH (1:10,000; Zen, China) used as an internal control. Protein was visualized by Image Quant LAS 4000 mini (GE, UK) in accordance with the manufacturer’s protocol. Densitometry analysis on the bands was performed using the NIH image J software and normalizing the data to total protein levels (Supplementary Figs 1 and 2).

### Statistical analysis

All quantitative values are expressed as the mean ± standard deviation (SD) and compared by Student’s t test or the Chi-squared test with SPSS 11.5 software (SPSS, USA). P values < 0.05 were considered to be statistically significant.

## Additional Information

**How to cite this article**: Liu, Z. *et al.* DNA Demethylation Rescues the Impaired Osteogenic Differentiation Ability of Human Periodontal Ligament Stem Cells in High Glucose. *Sci. Rep.* **6**, 27447; doi: 10.1038/srep27447 (2016).

## Supplementary Material

Supplementary Information

## Figures and Tables

**Table 1:** Forward and reverse primers for reverse transcription-polymerase chain reaction.

Gene	GenBank No.	Sequences(5′-3′)	Size
*DNMT1*	XM_011527774.1	Forward: ACCAAGAACGGCATCCTGTA	171
		Reverse: GCTGCCTTTGATGTAGTCGG	
*DNMT3a*	NM_175629.2	Forward: CTGGAAAAGGGAGGCTGAGA	228
		Reverse: TCGTACTCTGGCTCGTCATC	
*DNMT3b*	NM_006892.3	Forward: GGCCACCTTCAATAAGCTCG	197
		Reverse: GTTGCGTGTTGTTGGGTTTG	
*OPN*	NM_001040058.1	Forward: CAGTTGTCCCCACAGTAGACAC	127
		Reverse: GTGATGTCCTCGTCTGTAGCATC	
*ALP*	NM_000478.4	Forward: TAAGGACATCGCCTACCAGCTC	170
		Reverse: TCTTCCAGGTGTCAACGAGGT	
*OSX*	NM_001173467.2	Forward: GAGGTTCACTCGTTCGGATG	120
		Reverse: TGGTGTTTGCTCAGGTGGT	
*OCN*	NM_199173.5	Forward: CTCACACTCCTCGCCCTATT	115
		Reverse: CCTCCTGCTTGGACACAAA	
*GAPDH*	NM_002046.5	Forward: CTTTGGTATCGTGGAAGGACTC	132
		Reverse: GTAGAGGCAGGGATGATGTTCT	
