# Supplementary material for: DNA Demethylation Rescues the Impaired Osteogenic Differentiation Ability of Human Periodontal Ligament Stem Cells in High Glucose
Source: Sci Rep. 2016 Jun 8;6:27447. doi: 10.1038/srep27447 (PMC4897703; doi:10.1038/srep27447)
Supplement: Supplementary Information [file srep27447-s1.pdf]

DNA Demethylation Rescues the Impaired Osteogenic Differentiation Ability of Human Periodontal Ligament Stem Cells in High Glucose

Zhi Liu [1,2,3,┼], Tian Chen [2,3,4,┼], Wenhua Sun [2,3], Zongyi Yuan [1], Mei Yu [2,3], Guoqing Chen [2,3], Weihua Guo [2,3], Jingang Xiao [1,6,*] & Weidong Tian [2,3,5,*]

[1] Department of Oral and Maxillofacial Surgery, Hospital of Stomatology, Luzhou Medical College, Luzhou 646000, P.R. China

[2] National Engineering Laboratory for Oral Regenerative Medicine, West China Hospital of Stomatology, Sichuan University, Chengdu 610041, P.R. China

[3] State Key Laboratory of Oral Diseases, West China Hospital of Stomatology, Sichuan University, Chengdu 610041, P.R. China

[4] Department of Orthodontics, West China School of Stomatology, Sichuan University, Chengdu 610041, P.R. China

[5] Department of Oral and Maxillofacial Surgery, West China School of Stomatology, Sichuan University, Chengdu 610041, P.R. China

[6] Orofacial Reconstruction and Regeneration Laboratory, Hospital of Stomatology, Luzhou Medical College, Luzhou 646000, P.R. China

* Correspondence to: Jingang Xiao, Department of Oral and Maxillofacial Surgery, Hospital of Stomatology, Luzhou Medical College, No.2, Jiang Yang Nan Road, Luzhou 646000, PR China. Tel/fax: +86 0830 3165520. E-mail address: drxiaojg@sohu.com

Weidong Tian, Department of Oral and Maxillofacial Surgery, West China School of Stomatology, Sichuan University, No.14, 3rd Section, Renmin South Road Chengdu 610041, PR China. Tel/fax: +86 28 8550 3499. E-mail address: drtwd@sina.com

┼ The first two authors contributed equally to this work

Supplementary Figures

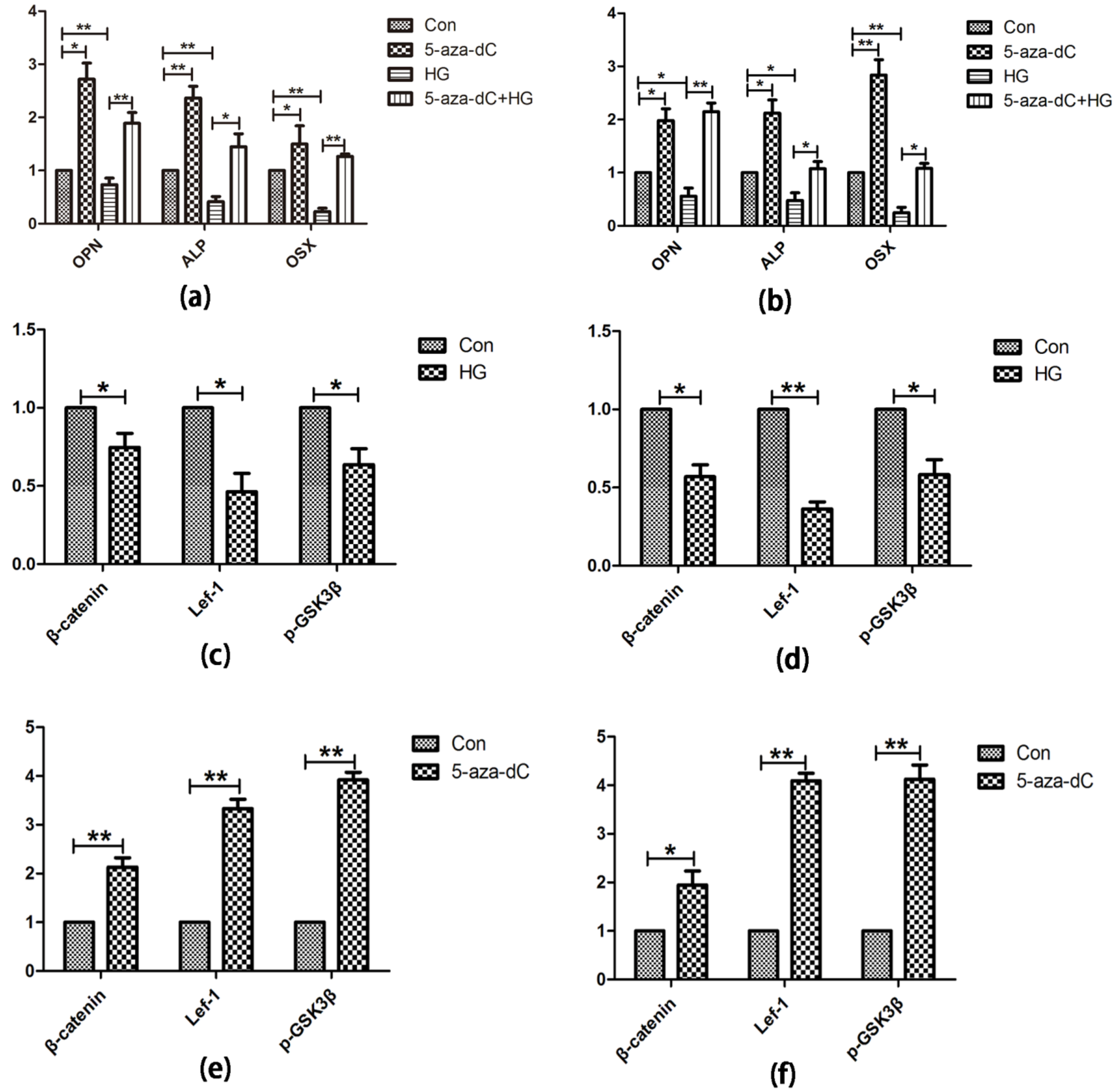

Supplementary Fig 1. Densitometry analysis on the western blotting bands. (a) Quantification of western blotting data of Fig 4g. (b) Quantification of western blotting data of Fig 4h. (c) Quantification of western blotting data of Fig 5a. (d) Quantification of western blotting data of Fig 5b. (e) Quantification of western blotting data of Fig 5c. (f) Quantification of western blotting data of Fig 5d. Densitometry analysis on the bands was performed using the NIH image J software and normalizing the data to total protein levels. GAPDH served as an internal control. *P <0.05, ** P < 0.01, n= 3.

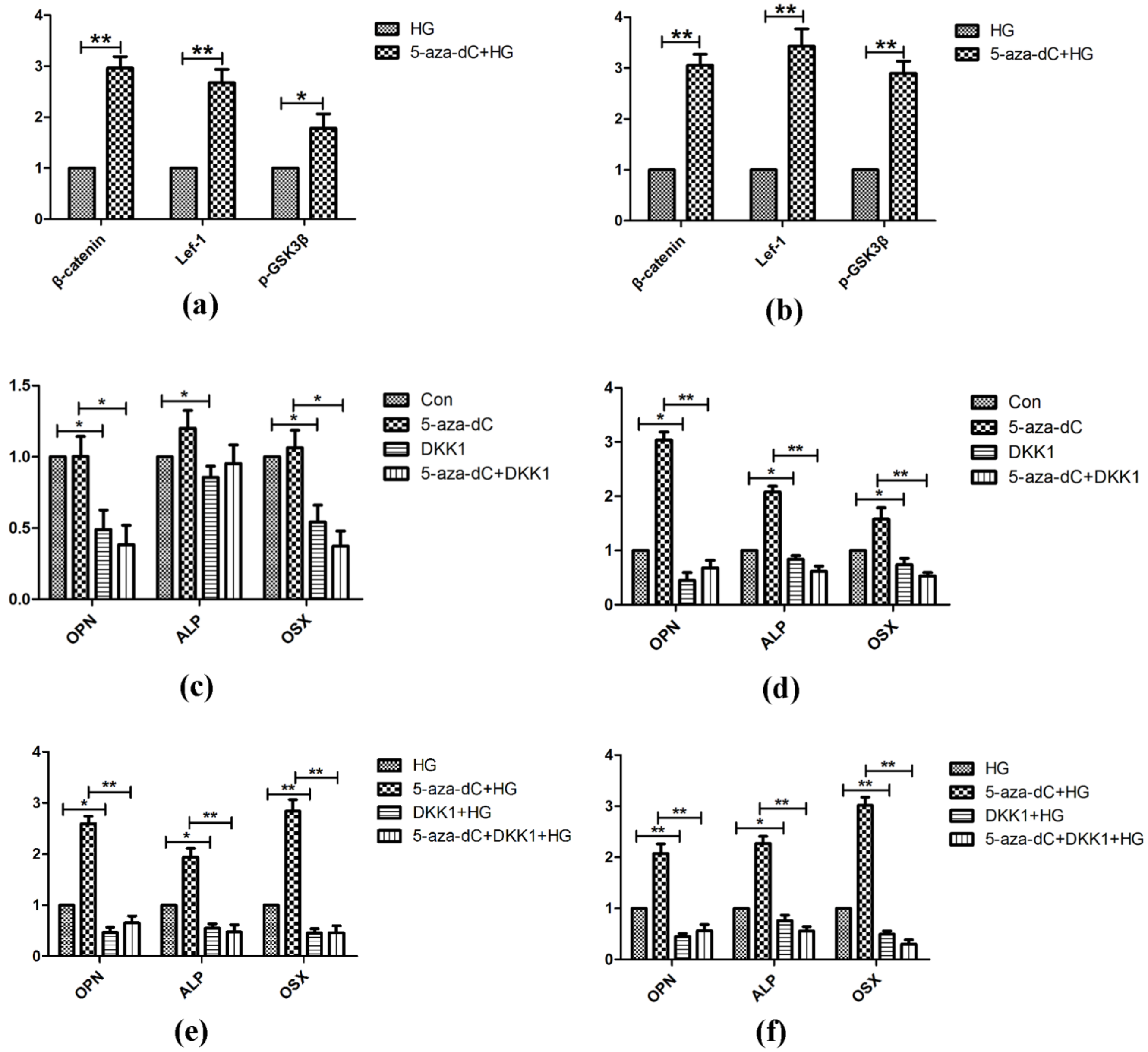

Supplementary Fig 2. Densitometry analysis on the western blotting bands. (a) Quantification of western blotting data of Fig 5e. (b) Quantification of western blotting data of Fig 5f. (c) Quantification of western blotting data of Fig 6a. (d) Quantification of western blotting data of Fig 6b. (e) Quantification of western blotting data of Fig 6c. (f) Quantification of western blotting data of Fig 6d. Densitometry analysis on the bands was performed using the NIH image J software and normalizing the data to total protein levels. GAPDH served as an internal control. *P <0.05, ** P < 0.01, n= 3.

Supplementary Tables

Table S1. Information of patients with healthy premolars extracted during orthodontic treatment

No.	Age	Gender	Health Condition	Diabetes or not
1	16	M	Good	No
2	14	M	Good	No
3	15	M	Good	No
4	14	M	Good	No
5	15	M	Good	No
6	17	M	Good	No
7	18	M	Good	No
8	15	M	Good	No
9	16	M	Good	No
10	14	M	Good	No
11	17	M	Good	No
12	18	M	Good	No

Information of patients with premolars extracted during orthodontic treatment in the West China Stomatology Hospital including patient age, gender, physical condition，and with or without diabetes. The names of each patient are replaced by numbers in order to protect personal privacy. Informed consent has been obtained from the patients involved.

Table S2. Bone microarchitecture in diabetic and non-diabetic rats

Structural Parameters	Control	Diabetic
BMD (g/cm^3)	0.67±0.11	0.51±0.19*
Tb.Th (μm)	0.13 ±0.01	0.12 ±0.02
Tb.N（1/mm）	3.41 ±0.13	2.38 ±0.22 *
Tb.Sp (μm)	0.15 ±0.07	0.26 ±0.03 *
BV/TV（%）	45.34 ±3.16	31.54 ±1.89 *

Bone microarchitecture analysis performed at alveolar bone at week 18 of diabetic rats and non-diabetic littermates. BMD, bone mineral density; Tb.Th, trabecular thickness; Tb.N, trabecular number; Tb.Sp, trabecular separation; BV/TV, bone volume on tissue volume. *P < 0.05, Diabetic vs. control (paired t test).
